# MicroRNAs: Game Changers in the Regulation of *α*-Synuclein in Parkinson's Disease

**DOI:** 10.1155/2019/1743183

**Published:** 2019-05-02

**Authors:** Liang Zhao, Zhiqin Wang

**Affiliations:** ^1^Department of Hematology, Xiangya Hospital, Central South University, Changsha, Hunan 410008, China; ^2^Department of Geriatrics, Xiangya Hospital, Central South University, Changsha, Hunan 410008, China; ^3^National Clinical Research Center for Geriatric Disorders, Xiangya Hospital, Central South University, Changsha, Hunan 410008, China

## Abstract

Parkinson's disease (PD) is the second most common neurodegenerative disorder. Its neuropathological hallmarks include neuronal loss in the substantia nigra pars compacta (SNpc) and the presence of Lewy bodies containing aggregates of *α*-synuclein (*α*-syn). An imbalance between the rates of *α*-syn synthesis, aggregation, and clearance can result in abnormal *α*-syn levels and contribute to the pathogenesis of PD. MicroRNAs (miRNAs) are endogenous single-stranded noncoding RNAs (∼22 nucleotides) that have recently emerged as key posttranscriptional regulators of gene expression. In this review, we summarize the functions of miRNAs that directly target *α*-syn. We also review miRNAs that indirectly impact *α*-syn levels or toxicity through different pathways, including those involved in the clearance of *α*-syn and neuroinflammation.

## 1. Introduction

Parkinson's disease (PD) is the second most common neurodegenerative disorder which affects 2-3% of the population ≥65 years of age [1]. Its cardinal motor features include tremor, rigidity, and bradykinesia [2]. The cause of PD is not fully understood. Both environmental and genetic factors contribute to the risk of developing PD [1, 3, 4, 5]. One of the neuropathological hallmarks of PD is neuronal loss in the substantia nigra pars compacta (SNpc), which causes striatal dopamine deficiency [1, 6]. Another key characteristic of PD is the formation of intracellular inclusions, namely, Lewy bodies, containing protein aggregates. In 1997, *α*-synuclein (*α*-syn), a protein with 140 amino acids, was identified as the most abundant protein in Lewy bodies. This followed the discovery that mutations in its gene, *SNCA*, which causes a monogenic autosomal dominant form of PD [7–10].

The molecular mechanisms through which abnormal *α*-syn aggregates contributing to neurodegeneration in PD remain unclear. *α*-Syn is thought to have a role in the regulation of neurotransmitter release, synaptic function, and plasticity [11]. Under healthy physical conditions, *α*-syn exists predominantly as a monomer. During pathogenesis, soluble *α*-syn monomers may initially form oligomers, including low-molecular-weight species (such as dimers, trimers, and tetramers) and high-molecular-weight species (such as spherical, chain-like, and annular structures). These structures progressively combine to form small protofibrils which further aggregate into large, insoluble fibrils [12]. The fibrillar forms of *α*-syn are detected mainly in Lewy bodies, which are localized in the neuronal cell body. Oligomeric aggregates are usually found in axons and presynaptic terminals, forming Lewy neurites [1].

Prefibrillar oligomers may represent the toxic form of *α*-syn [13, 14], whereas Lewy bodies are thought to reflect an attempt by the neurons to isolate and/or convert toxic *α*-syn oligomers to fibrils, which are stable, less dynamic structures that exhibit reduced toxicity [11]. Conversion of *α*-syn to the toxic oligomeric form may be modulated by many factors, including oxidative stress [15], post-translational modifications [16], interactions with lipids or small molecules [11], and the concentration of *α*-syn [11].

An imbalance between the rates of *α*-syn synthesis, aggregation, and clearance can result in an abnormal level of *α*-syn and thus the formation of toxic oligomeric and fibrillar species [17]. Indeed, increased expression of *α*-syn due to *SNCA* duplication was found to be a cause of familial PD [18, 19]. Biological processes that clear *α*-syn monomers and aggregates include direct proteolysis [20], binding to molecular chaperones [21], and the proteasome [22] and autophagy pathways [23, 24]. Dysfunction of these processes may also contribute to PD [23, 25].

MicroRNAs (miRNAs) are endogenous single-stranded noncoding RNAs (∼22 nucleotides) that have recently emerged as key posttranscriptional regulators of gene expression [26, 27]. Regions of the genome that encode miRNAs are transcribed in the cell nucleus, producing long primary miRNAs (pri-miRNAs), which are up to several kilobases in length. The RNase III enzyme Drosha converts pri-miRNAs into 70–100 nucleotide (nt) stem-loop structures called pre-miRNAs. Pre-miRNAs are transported to the cytoplasm and cleaved by the enzyme dicer into small, ∼22 nt, miRNA-miRNA complex intermediates. Then, the RNA duplex binds to an argonaute (AGO) protein, and one of the strands is removed, resulting in the mature RNA-induced silencing complex (RISC). RISC can suppress the translation and/or promote the degradation of target mRNAs by binding to their 3'-untranslated regions (3′-UTRs). miRNAs are abundant in the nervous system and have key roles in maintaining efficient brain function [28–32].

Considering the importance of *α*-syn in the pathogenesis of PD and the fact that miRNAs are involved in the regulation of *α*-syn [33, 34], we have summarized the functions of miRNAs that directly target *α*-syn. We also reviewed miRNAs that indirectly impact the level or toxicity of *α*-syn through various pathways, including those involved in the clearance of *α*-syn and neuroinflammation.

## 2. miRNAs That Directly Bind to the 3′-UTR of *α*-Syn and Their Roles in PD

### 2.1. miR-7

**Figure 1:**
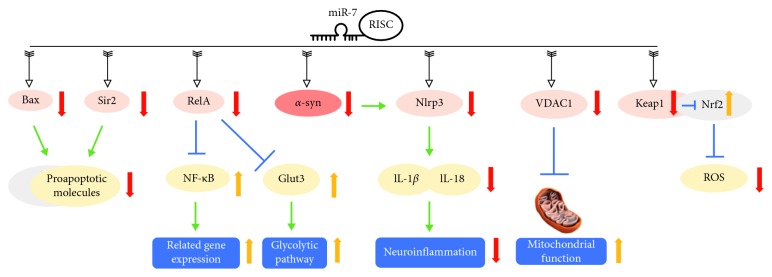
miR-7 playing a neuroprotective role in PD. miR-7 has multiple direct targets, including *α*-syn, Bax, Sir2, RelA, Nlrp3, VDAC1, and Keap1. By directly targeting the 3′-UTR of *α*-syn, miR-7 significantly reduces *α*-syn expression. miR-7 downregulates Nlrp3 through direct targeting and inhibiting *α*-syn upstream, resulting in decreased levels of IL-1*β* and IL-18 and alleviating neuroinflammation. In another pathway, miR-7 inhibits Bax and Sir2 expression, preventing apoptosis. Additionally, miR-7 downregulates RelA, which causes downstream upregulation of NF-*κ*B and Glut3, resulting in the activation of gene expression in glycolysis and NF-*κ*B pathways. Moreover, by directly targeting VDAC1, miR-7 activates mitochondrial function. miR-7 also reduces reactive oxygen species (ROS) via the Keap1-Nrf2 pathway. All of the above contribute to the neuroprotective roles of miR-7 in PD.

In 2009, Junn et al. utilized public prediction algorithms to identify miRNAs that could regulate *α*-syn expression, and miR-7 was the only candidate matching all common predictors [35]. MiR-7 downregulated *α*-syn expression by directly binding to the 3′-UTR of *α*-syn mRNA, a role which was confirmed by a firefly luciferase reporter assay in human dopaminergic (DAergic) neuroblastoma SH-SY5Y cells. Moreover, miR-7 co-localized with *α*-syn in neurons, fitting with the localization pattern of a certain miRNA and its target mRNA. In the MPTP (1-methyl-4-phenyl-1,2,3,6-tetrahydropyridine) mouse model, MPTP intoxication upregulates *α*-syn expression while reducing miR-7 expression in the ventral midbrain [35]. In the mouse SNpc, a knockdown of miR-7 resulted in obvious *α*-syn overexpression [36]. Clinically, PD patients have significantly lower miR-7 levels in the SNpc [36]. All these data suggest that miR-7 regulates *α*-syn expression and therefore is associated with the pathogenesis of PD (Figure 1).

Besides directly targeting *α*-syn, other neuroprotective effects of miR-7 have been widely studied (Figure 1).

Bax and sirtuin 2 (Sir2) are two important proteins that can activate proapoptotic molecules in the 1-methyl-4-phenylpyridinium (MPP+)-induced model [37, 38]. Bax and Sirt2 were shown to be direct targets of miR-7 [39]. Decreased expressions of proapoptotic molecules downstream of Bax and Sirt2 were involved in the neuroprotective property of miR-7 [39].

NF-*κ*B is a ubiquitously expressed transcription factor that regulates gene expression and is involved in a variety of processes, such as inflammation and apoptosis [40]. In PD models, RelA mediates MPP+-induced suppression of NF-*κ*B activity, which is essential for MPP+-induced cell death. miR-7 directly targets RelA mRNA, thus protecting DAergic neurons from toxicity by suppressing RelA expression [41]. In a subsequent study, knockdown of RelA through the overexpression of miR-7 led to the increased expression of Glut3 [42], the major neuronal cell surface glucose transporter [43]. Glut3 silencing, the presence of a low glucose medium, or treatment with a glycolytic pathway inhibitor, diminishes the protective effects of miR-7 against MPP+, indicating that a functional glycolytic pathway is required for its protective effects [42]. Voltage-dependent anion channel 1 (VDAC1) was found to be another direct target of miR-7 [44]. VDAC1 is an integral protein of the mitochondrial outer membrane and is involved in the response to cytotoxic stimuli, which ultimately trigger cell death [45]. The miR-7 protective effects are partially exerted through the promotion of mitochondrial function by targeting VDAC1 expression [44].

Nuclear factor E2-related factor 2 (Nrf2) is a key transcription factor, which activates the expression of several antioxidant and phase II detoxifying genes for protection against various stressors, including reactive oxygen species (ROS) [46]. In normal physiological conditions, Nrf2 is mainly localized in the cytoplasm in a complex with an inhibitory protein, Kelch-like ECH-associated protein 1 (Keap1) [47]. Kabaria et al. showed that miR-7 could directly target Keap1 and results in increased Nrf2 activity. Through this Keap1-Nrf2 axis, miR-7 reduces cellular ROS, exerting its cytoprotective effects [48]. Interestingly, miR-7-mediated translational suppression of *α*-syn can also be relieved by MPP+-mediated mitochondrial ROS [49].

Mammalian target of rapamycin complexes (mTORC1/2) serves as indispensable regulators of cell metabolism, growth, and survival [50]. Stress-activated protein kinase/c-Jun NH2-terminal kinase (SAPK/JNK) is a kinase with a central role in neuronal microtubule stability [51]. Suppression of mTOR and SAPK/JNK signaling pathways contributes to MPP+-induced cell death [52, 53]. Fragkouli and Doxakis showed that miR-7 could protect neurons by recovering the activation of mTOR and SAPK/JNK signaling pathways [53].

miRNAs work as regulators of their mRNA targets. Intriguingly, it has been recently shown that miRNAs themselves could be targeted and regulated by RNA molecules. These miRNA sponge transcripts, the so-called competing endogenous RNAs (ceRNAs), de-repress all target genes of the respective miRNA family [54]. CiRS-7 (circular RNA sponge for miR-7) was identified as a miR-7 sponge, which can strongly inhibit miR-7 activity, resulting in increased levels of miR-7 targets, including *α*-syn [54]. The role of ciRS-7 in PD requires further investigation. Recently, circular SNCA RNA (circSNCA) was reported as another miR-7 sponge [55]. Pramipexole (PPX), a dopamine receptor agonist with proven efficacy in the treatment of PD, downregulates circSNCA in a MPTP model, leading to miR-7 upregulation and *α*-syn downregulation, resulting in reduced cell apoptosis [55]. More comprehensive studies regarding the miR-7 sponge are necessary for a complete understanding of the *α*-syn regulatory network.

Regulation of autophagy [56] and *α*-syn-induced inflammation [57, 58] also contributes to the neuroprotective properties of miR-7, and these will be discussed later in this review.

### 2.2. miR-153

miR-153 is another miRNA that posttranscriptionally regulates *α*-syn expression [59]. Junn et al. predicted that miR-153 could bind to *α*-syn mRNA based on prediction algorithms [35]. Doxakis demonstrated that miR-153 can directly target the *α*-syn 3′-UTR and downregulate its mRNA and protein levels [59]. It is intriguing that miR-7 and miR-153 may utilize different kinetics to regulate *α*-syn. That is, miR-7 has a stronger effect on *α*-syn protein translation inhibition, while miR-153 transiently impacts mRNA degradation [59]. Like miR-7, miR-153-mediated translational suppression of *α*-syn can also be relieved by MPP+-mediated mitochondrial ROS [49]. In addition, miR-153 may protect cortical neurons from MPP+-induced toxicity by preserving the activation of mTOR and SAPK/JNK signaling pathways, while attenuating MPP+-induced activation of p38 MAPK [53].

Nucleotides 459–465 of the *SNCA* 3′-UTR were predicted to be the target sequence for miR-153 [60]. A rare variation in the 3′-UTR of *SNCA*, 464 C>A, was reported in a PD patient [60]. This variation is able to disturb the hybrid structure between miR-153 and *α*-syn mRNA, thus attenuating the inhibiting ability of miR-153 [60]. Whether this variant is a rare cause of PD requires further investigation.

### 2.3. miR-34b and miR-34c

miR-34b and miR-34c directly target *α*-syn, inhibiting its expression and aggregation formation [61]. An SNP in the 3′-UTR of *SNCA*, namely, rs10024743, is able to reduce miR-34b-mediated repression of *α*-syn expression [61]. The association of this polymorphism with PD risk needs to be clarified. miR-34c-5p was found to be decreased in the amygdala, frontal cortex, substantia nigra, and cerebellum of PD patients in both clinical (motor) stages (Braak stages 4 and 5) and the premotor stages (stages 1–3), compared with that of control individuals [62]. Depletion of miR-34b or miR-34c in vitro results in reduced cell viability, which is accompanied by mitochondrial dysfunction, elevation of cellular ROS, and downregulation of DJ1 and Parkin, two proteins associated with the familial forms of PD [62].

The adenosine A_2A_ receptor (A_2A_R) is a G protein-coupled receptor. In the brain, A_2A_Rs are highly enriched in striatal GABAergic medium spiny neurons, which help control voluntary movement [63]. A_2A_Rs protein levels are increased in the putamen of PD cases, in both motor and premotor disease stages [64]. miR-34b has been shown to directly target A_2A_Rs [64], but the role of this interaction in PD requires further investigation.

With an increase in nonionizing radiation arising from both environmental and manmade sources, exposure to electromagnetic fields (EMFs) and their subsequent pathogenic effects have become a growing concern [65]. Extremely low-frequency (0 Hz to 100 kHz) magnetic fields (ELF-MFs) have been associated with an increased risk of neurodegenerative disorders, such as Alzheimer's disease (AD), whereas an univocal association with PD is still lacking [66]. Consales et al. found that ELF-MFs downregulated miR-34b and miR-34c in human DAergic neuroblastoma SH-SY5Y cells. This effect is caused by hypermethylation of the CpG island within the miR-34b/c promoter [67]. ELF-MFs exposure stimulated *α*-syn expression via miR-34b/c downregulation and oxidative stress, demonstrating that environmental factors affect miR-34b/c and may be involved in the pathogenesis of PD [67].

### 2.4. miR-214

miR-214 is another miRNA that can directly target *α*-syn [68]. In the MPP+-induced PD model, Wang et al. found downregulation of miR-214 and upregulation of *α*-syn, indicating a neuroprotective role for miR-214 [68]. The effects of miR-214 in PD warrant additional studies.

## 3. miRNAs That Indirectly Impact *α*-Syn without Binding to the *α*-Syn 3′-UTR

**Figure 2:**
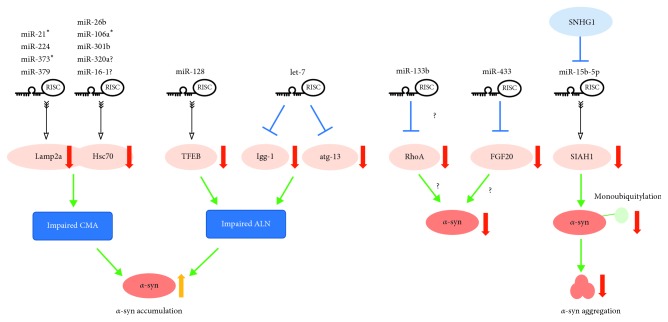
miRNAs that indirectly impact *α*-syn without binding to its 3′-UTR. Several miRNAs cause impairment of the chaperone-mediated autophagy (CMA) and autophagy-lysosome network (ALN) pathways, leading to *α*-syn accumulation. Lamp2a in the CMA pathway is targeted by miR-21^∗^, miR-224, miR-373^∗^, and miR-379, while Hsc70 is targeted by miR-26b, miR-106a^∗^, miR-301b, and probably miR-320a and miR-16-1. In the ALN, TFEB, which is necessary for lysosomal biogenesis and function, is directly targeted and reduced by miR-128. Let-7, on the other hand, can suppress lgg-1 and atg-13. Therefore, miR-128 and let-7 contribute to PD pathogenesis by impairing the ALN. On the other hand, SIAH1, a monoubiquitylation modifier of *α*-syn, is inhibited by miR-15b-5p, leading to decreased *α*-syn aggregation. Moreover, lncRNA SNHG1 could directly bind miR-15-5p and repress miR-15-5p expression. The roles of miR-133b and miR-433 are controversial. miR-133b may inhibit *α*-syn expression via inhibition of RhoA, while miR-433 may suppress *α*-syn expression via FGF20 inhibition.

**Figure 3:**
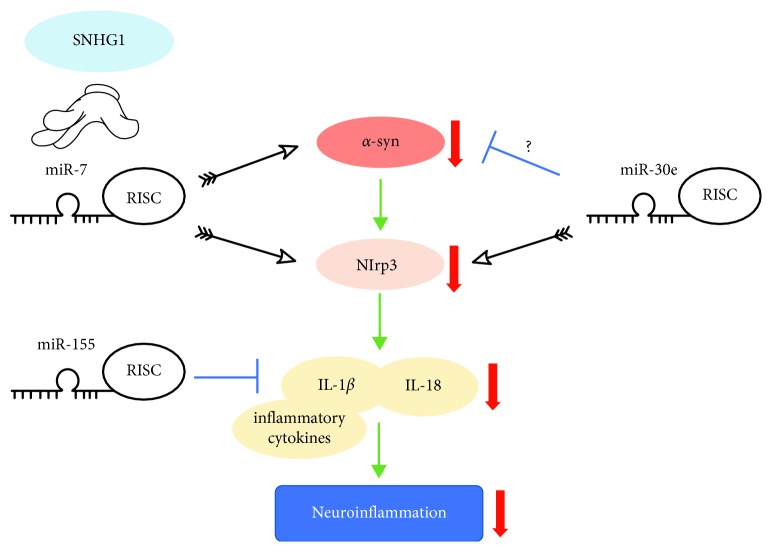
miRNAs that regulate *α*-syn-induced neuroinflammation. Nlrp3 is a key component of a cytoplasmic multiprotein called the “inflammasome,” which can critically control the activity of IL-1*β* and IL-18. As mentioned in Figure 1, miR-7 alleviates neuroinflammation through the *α*-syn/Nlrp3 axis. SNHG1 competes with Nlrp3 for miR-7 and aggravates neuroinflammation. miR-30e can also attenuate inflammatory cytokines by directly targeting Nlrp3 and possibly inhibiting *α*-syn expression in an indirect way. miR-155 may also inhibit *α*-syn-induced proinflammatory responses.

Besides directly targeting *α*-syn, some miRNAs exert their effects in PD by indirectly impacting the level or toxicity of *α*-syn through a variety of pathways, including those involved in the clearance of *α*-syn (Figure 2) and neuroinflammation (Figure 3).

### 3.1. miRNAs that Regulate the Clearance of *α*-Syn via CMA

As mentioned above, the clearance of *α*-synuclein monomers and aggregates occurs through several pathways, including the chaperone-mediated autophagy (CMA) pathway [69]. In this pathway, a pentapeptide motif (KFERQ) presented in the protein is recognized by the heat shock cognate protein 70 (Hsc70) chaperone and internalized into the lysosome by the lysosomal-associated membrane protein 2a (Lamp2a) membrane receptor [69]. *α*-Syn contains a pentapeptide sequence (VKKDQ) that is consistent with a CMA recognition motif, by which *α*-syn is selectively translocated into lysosomes for degradation [23]. Inhibition of CMA through downregulation of Lamp2a protein levels leads to *α*-syn accumulation [70, 71] and neurodegeneration [72]. Lamp2a and Hsc70 proteins are decreased in the SNpc and amygdala in PD brains compared with both age-matched controls and AD patients [71] (Figure 2).

Alvarez-Erviti et al. used the miRBase target database to predict which miRNAs directly target the 3′-UTR of Lamp2a or Hsc70 mRNA in humans. The luciferase reporter assay in SH-SY5Y cells demonstrated that four miRNAs (hsa-miR-21^∗^; hsa-miR-224; hsa-miR-373^∗^; and hsa-miR-379) target the 3′-UTR of Lamp2a, while three miRNAs target the 3′-UTR of Hsc70 (hsa-miR-26b; hsa-miR-106a^∗^; and hsa-miR-301b). Transfection of these miRNAs also decreases endogenous Lamp2a and Hsc70 protein levels, resulting in significant *α*-syn accumulation. The analysis of PD brains confirmed that six of these miRNAs, including hsa-miR-21^∗^, hsa-miR-224, hsa-miR-373^∗^, hsa-miR-26b, hsa-miR-106a^∗^, and hsa-miR-301b, were significantly increased in the SNpc [73] (Figure 2).

The possibility that miR-21 could reduce Lamp2a and increase *α*-syn levels was confirmed by a second study using SH-SY5Y cells and the MPTP-treated PD mouse model [74]. This study also suggested that geniposide (GP) had a neuroprotective effect against MPP+ by reducing miR-21, increasing Lamp2a, and thus decreasing *α*-syn [74]. GP is a major iridoid glycoside extracted from the fruit of *Gardenia jasminoides*, which is a widely used herb in traditional Chinese medicine [75]. The study by Su et al. showed that the miR-21/Lamp2a/*α*-syn axis was a promising target for PD treatment [74].

Two additional miRNAs, miR-320a [76] and miR-16-1 [77], were reported to be direct regulators of Hsc70, promoting *α*-syn aggregation in SH-SY5Y cells that overexpress *α*-syn (Figure 2). However, whether these miRNAs can directly target the 3′-UTR of Hsc70 needs to be confirmed, as a luciferase reporter assay was not conducted in these two studies [76, 77].

### 3.2. miRNAs that Regulate the Clearance of *α*-Syn via the ALN

The autophagy-lysosome network (ALN) is another pathway that degrades *α*-syn under physiological conditions [22, 78, 79]. miR-7 has been shown to facilitate the degradation of *α*-syn and its aggregates by promoting autophagy, but the detailed mechanism remains unclear [56]. Studies regarding other miRNAs have provided some interesting information about how they affect *α*-syn degradation via the ALN.

As a master regulator of the ALN, transcription factor EB (TFEB) is inhibited by mTOR signaling [80, 81, 82]. Enhancement of TFEB function has been shown to stimulate ALN function and promote protein clearance [80]. TFEB function is impaired in the rat PD model, as well as in the human PD midbrain, resulting in the accumulation of *α*-syn oligomers, development of DAergic neuron pathology, and cell death [83]. miR-128 has been shown to target TFEB [80]. In DAergic neurons, miR-128 repression of TFEB caused an increase in vulnerability to *α*-syn toxicity [83] (Figure 2).

Let-7 is another miRNA reported to regulate *α*-syn expression via alterations in ALN [84]. Let-7 is a highly conserved miRNA that has been reported to repress several oncogenes by affecting cell cycle, cell differentiation, and apoptotic pathways [85]. Let-7 miRNA is increased in a *C. elegans* model of PD, where the human *α*-syn protein is expressed [84]. Knockdown of let-7 miRNA leads to reduced expression of *α*-syn protein and increased levels of lgg-1 and atg-13 [84]. Lgg-1 is an ortholog of *Saccharomyces cerevisiae* Atg8p and mammalian MAP-LC3, which is required for the degradation of cellular components [86, 87]. The autophagy-related gene, aatg-13, is required for autophagosome formation [88]. These results indicated that the knockdown of let-7 may reduce *α*-syn level via activation of ALN (Figure 2).

### 3.3. Controversial Roles of miR-133b and miR-433

miR-133b has been shown to decrease *α*-syn expression and ameliorate the MPP+ -induced increase of *α*-syn in vitro [89]. The evidence that *α*-syn is a direct target of miR-133b has not been reported. Niu et al. attributed *α*-syn downregulation to miR-133 inhibition of RhoA [89, 90], a Rho family member that plays important roles in apoptosis and suppresses neurite extension [91], but additional studies are required to confirm this pathway's involvement. In fact, the role of miR-133b in PD is controversial. Kim et al. found that miR-133b is specifically enriched in the midbrain under healthy conditions and is deficient in both PD patient samples (*n*=3) and in mouse models, indicating that reduced miR-133b may be involved in the pathogenesis of PD [92]. However, Heyer et al. failed to observe abnormal DAergic neurons numbers or the expected general midbrain or striatum morphology in the miR-133b null mouse [93]. In another study, miR-133b levels were found unaltered in five sporadic PD brains compared to eight healthy controls [94].

It was reported that a single-nucleotide polymorphism (SNP), rs12720208, in the 3′-UTR of fibroblast growth factor 20 (FGF20) mRNA, a member of the FGF family [95], disrupts a binding site for miRNA-433, increasing the level of FGF20 in vitro and in vivo [96]. In both SH-SY5Y cells and PD brains, this FGF20 increase is correlated with increased *α*-syn levels [96]. However, these results need further validation since subsequent reports found no evidence of an association between FGF20 variability and PD risk [97, 98]. Also, no relationship could connect the rs12720208 genotype, FGF20, and *α*-syn protein levels [97] (Figure 2).

### 3.4. miR-15b-5p Inhibits *α*-Syn Aggregation

miR-15b-5p was reported to regulate *α*-syn aggregation and toxicity rather than expression levels [99] (Figure 2). Seven in absentia homolog 1 (SIAH1) is an E3 ubiquitin ligase, which plays an important role in promoting *α*-syn monoubiquitylation and aggregation, contributing to the formation of Lewy bodies [100]. SIAH1 was found to be a direct target of miR-15b-5p. Overexpression of miR-15b-5p alleviates *α*-syn aggregation and cell apoptosis in SH-SY5Y neurons overexpressing *α*-syn [99]. In addition, the long noncoding RNA (lnRNA) and small nucleolar RNA host gene 1 (SNHG1) directly bind to miR-15-5p and repress its expression [99]. It would be interesting to study the role of the SNHG1/miR-15b-5p/SIAH1 axis in PD.

### 3.5. miRNAs that Regulate *α*-syn-induced Inflammation

miR-155, which is significantly upregulated in an in vivo model of PD, was reported to regulate *α*-syn-induced inflammation [101]. In a miR-155 null mouse model, proinflammatory responses to *α*-syn were reduced and *α*-syn-induced neurodegeneration was blocked [101]. In primary microglia from miR-155^−/−^ mice, there is a markedly reduced inflammatory response to *α*-syn fibrils, which is restored following the treatment with a synthetic mimic of miR-155 [101]. These data suggest that miR-155 is involved in the PD pathogenesis, in part due to its role in the regulation of microglial responses to *α*-syn (Figure 3).

As we discussed above, miR-7 can directly target *α*-syn and thus protect neurons [35, 59]. It has recently been found that miR-7 has a protective role in PD mice by inhibiting neuroinflammation [57]. Nod-like receptor protein 3 (Nlrp3) is one component of a cytoplasmic multiprotein called the “inflammasome”, which can critically control the activity of IL-1*β* and IL-18 [102]. *α*-Syn activates the Nlrp3 inflammasome through microglial endocytosis and subsequent lysosomal cathepsin B release [57]. miR-7 can target Nlrp3 directly, inhibiting *α*-syn/Nlrp3 axis neuroinflammation [57] (Figure 3).

Another miRNA, miR-30e, has recently been found to directly target Nlrp3 [103]. miR-30e agomir administration attenuates the marked increase of inflammatory cytokines, such as TNF-*α*, COX-2, and iNOS in the SNpc of MPTP-induced PD mice. miR-30e also alleviates the upregulation of *α*-syn, but not by directly targeting it [103]. In addition, Cao et al. proved that SNHG1 can compete with Nlrp3 for miR-7 (Figure 3). Upregulation of SNHG1 leads to activation of the Nlrp3 inflammasome [58]. These findings suggest that miRNAs are involved in the regulation of inflammation in PD.

## 4. Conclusions

PD is the second most common neurodegenerative disease. The pathogenesis of PD is not fully understood. Currently, there is no effective etiological treatment for PD. Epigenetics alterations, including miRNAs, play important roles in neurological disorders. Abnormal *α*-syn expression levels and aggregates contribute to the neurodegeneration in PD, but the molecular mechanism remains unclear. Several miRNAs can directly target *α*-syn and exert their neuroprotective effects. Some of them, such as miR-7, can also regulate pathways independent of *α*-syn. Additionally, several miRNAs indirectly impact *α*-syn levels without binding to the *α*-syn 3′-UTR, via impact on the CMA pathway or the ALN. Some miRNAs participate in PD pathogenesis by regulating *α*-syn-mediated toxicity or neuroinflammation. Further studies are required to understand the complete network between miRNAs, *α*-syn, and PD, in order to develop effective miRNA-based therapies.
